# Russian heritage language development in narrative contexts: Evidence from pre- and primary-school children in Norway, Germany, and the UK

**DOI:** 10.3389/fpsyg.2023.1101995

**Published:** 2023-02-09

**Authors:** Yulia Rodina, Alexandra Bogoyavlenskaya, Natalia Mitrofanova, Marit Westergaard

**Affiliations:** ^1^Department of Language and Culture, UiT The Arctic University of Norway, Tromsø, Norway; ^2^Department of Language and Literature, NTNU Norwegian University of Science and Technology, Trondheim, Norway

**Keywords:** lexical development, Russian heritage language, oral narratives, individual factors, Germany, Norway, the UK

## Abstract

The present study aims at obtaining a comprehensive picture of language development in Russian heritage language (RHL) by bringing together evidence from previous investigations focusing on morphosyntax and global accent as well as from a newly conducted analysis of a less-studied domain–lexical development. Our investigation is based on a narrative sample of 143 pre- and primary-school bilinguals acquiring RHL in Norway, Germany, and the United Kingdom. We performed a multiple-way analysis of lexical production in RHL across the different national contexts, across both languages (heritage and societal), also comparing bilinguals and monolinguals. The results revealed a clear and steady increase with age in narrative length and lexical diversity for all bilingual groups in both of their languages. The variation in lexical productivity as well as the differences between the bilingual groups and between bilinguals and monolinguals were attributed to input factors with language exposure in the home and age of starting preschool as the major predictors. We conclude that, overall, the results from lexical, grammatical, and phonological acquisition in RHL support the view that having longer exclusive or uninterrupted exposure to a heritage language in early childhood is beneficial for its development across domains.

## 1. Introduction

Russian heritage language (RHL) has a prominent place in the empirical landscape of heritage language research. In the past two decades, a large number of studies have appeared around the world reporting data from child and adult heritage speakers of Russian with different societal majority languages (English, German, Hebrew, Norwegian, Finnish, Swedish, Latvian, Spanish, and Dutch among others). Thus, to date there is considerable knowledge about the linguistic behavior and competence in RHL at early and later stages of language development (Dieser, 2009; Polinsky, and Maria., 2008; Laleko, 2010, 2022; Schwartz et al., 2015; Brehmer and Kurbangulova, 2017; Rodina, 2017; Gagarina et al., 2021; Krüger, 2021; Meir and Janssen, 2021; Otwinowska et al., 2021; among others). The available observations come primarily from specific case studies. Large-scale investigations studying RHL development across a wider age range and a larger number of children are scarce. To fill in this gap, in the current study, we investigate heritage language development in pre- and primary-school children between the ages of 3 and 10 based on data obtained in a large-scale project focusing on the grammatical and phonological (global accent) development in RHL in Norway, Germany, and the United Kingdom (UK) (Mitrofanova et al., 2018, 2022; Rodina et al., 2020; Kupisch et al., 2021).^1^

The in-depth investigation of grammatical gender in these studies revealed that bilinguals in different national contexts developed fine-grained sensitivity to grammatical gender cues in Russian, which ensured their successful acquisition of this property. It was also evident that pre- and primary-school bilinguals as well as Russian monolinguals apply the same mechanisms and display the same developmental patterns in the acquisition of gender. Furthermore, in a subset of the data collected in Germany, we observed a shift from sounding more accented in the majority language during preschool to sounding more accented in RHL in primary school years (due to a change in exposure patterns characterized by a steady increase in the exposure and use of the majority language). Both the acquisition of gender and global accent patterns in RHL were found to be affected by several background variables, including family type, age of starting preschool, and exposure to RHL instruction as the most important ones.

To obtain a comprehensive picture of language development in RHL, in the present study, we focus on a less-studied domain–lexical development. We perform a multiple-way comparison of oral language samples of 143 German-Russian, Norwegian-Russian, and English-Russian bilinguals aged 3–10 as well as 31 Russian-speaking monolingual peers. Lexical production patterns are assessed in both of the bilinguals’ languages with an ecological language procedure, narrative storytelling, which, in contrast to vocabulary tests, taps into the ability to use vocabulary in real-life situations. The oral language samples in the present study were obtained using narrative elicitation material in the Multilingual Assessment Instrument for Narratives (MAIN) (Gagarina et al., 2012). To investigate vocabulary growth, we employ two widely used measures: total number of words (TNW) and the number of different words (NDW). We also explore the relationship between lexical productivity measures and the individual background factors which were found to be important predictors of development across different linguistic domains (Lloyd-Smith et al., 2020).

## 2. Previous research on lexical development in RHL

Much of the existing evidence identifies lexicon as a domain of major deficits in heritage language speakers across different languages including Russian (for an overview, see Unsworth, 2013; Scontras et al., 2015). Specifically, due to the distributed and context-specific nature of bilingual language learning, bilingual children are typically found to score below age-appropriate norms for monolingual children on tasks of receptive vocabulary, such as the Peabody Picture Vocabulary Test (PPVT) (Pearson et al., 1993, 1997; Dunn and Dunn, 2007). Differences in vocabulary development and lexical retrieval between heritage and monolingual children are reported in various other studies (e.g., Yan and Nicoladis, 2009; Silvén et al., 2014; Jia and Paradis, 2015). Importantly, a comparison of lexical development in younger and older bilingual children typically reveals a rapid age-related growth of vocabulary in the majority language, but a stabilization or stagnation of vocabulary development in the heritage language as a function of the shift in exposure, causing a shift in language dominance (Gathercole and Thomas, 2009; Bialystok et al., 2010; Sheng et al., 2011). This shift is known to be unique to Heritage Speakers’ (HS) acquisition trajectory and usually occurs when the child starts formal education (Montrul, 2016). The onset of schooling (taking place as early as age 5 in some countries) is characterized by an increase in input and use of the societal majority language and a corresponding decrease in input and use of the HL characteristic of the home environment. The acquisition of literacy in the majority language further contributes to this shift in the linguistic environment of HSs. Such a shift is shown to affect all linguistic domains, including vocabulary. As discussed below, this shift shapes bilinguals’ lexical development in RHL and will be important for the discussion of the results of the current study in the (Section “5. Discussion”).

Previous research on RHL addressed certain aspects of vocabulary acquisition based on narrative, experimental, and longitudinal data (Bar-Shalom and Zaretsky, 2008; Gagarina et al., 2014; Klassert et al., 2014; Ringblom and Dobrova, 2019; Makarova and Terekhova, 2020; Montanari et al., 2020; Czapka et al., 2021). Several of the studies have been conducted in Germany, where Russian is one of the most frequently spoken and intensively investigated HLs. For example, Klassert et al. (2014) investigated a rarely addressed question of whether nouns are more vulnerable in bilingual acquisition than verbs. Their comparison of naming abilities for nouns and verbs in three age groups of German-Russian bilinguals (4;0–4;11, 5;0–5;11, and 6;0–6;11) and four age groups of Russian and German monolinguals (3;6–3;11, 4;0–4;11, 5;0–5;11, and 6;0–6;11) revealed a more pronounced naming deficit for nouns than for verbs, since bilinguals performed consistently below the younger monolingual children in noun naming. The higher vulnerability of nouns has been attributed to the reduced input for bilingual children as well as to the different distributions of nouns and verbs in the input. Of relevance to the present study is another central observation of Klassert et al. (2014): While verb naming developed at a similar rate in Russian and German, there was a stronger growth in noun naming in German than in Russian in 5- and 6-year-olds. This is explained by a combination of language internal and language external factors, such as the availability and saliency of nouns and verbs in the input of bilingual children and most importantly the shift in language dominance toward German at around age 5.

More recently, Montanari et al. (2020) and Czapka et al. (2021) investigated the developmental trajectories of pre- and primary-school German-Russian bilinguals. Both are comparative studies of lexical development in Russian and Turkish HLs, showing that the migrant community characteristics mediate HL acquisition in important ways. In the longitudinal sample of Russian 2–4-year-olds (*n* = 70), Czapka et al. (2021) observe a significant growth of expressive vocabulary over the course of four testing times. The children’s societal language, German, was not tested, but importantly, a later age of onset of German as well as more HL input from siblings were found to be significant predictors of vocabulary size in RHL. In contrast, the expressive vocabulary of German-Russian bilinguals (*n* = 113, age range 6–10) in Montanari et al. (2020) failed to progress in the timespan of four primary school years, which was interpreted as a sign of attrition. Yet, a picture-naming task revealed that the expressive as well as the receptive vocabularies were already well-developed in the youngest children in this study. A considerably large vocabulary size in RHL was found to correlate with several characteristics of the Russian-speaking community, such as the mothers’ proficiency in the HL, parental level of education (university degree), place where the highest level of education was obtained (the country of origin) as well as HL support from associations or school classes. At the same time, there was no shift toward German detected in this bilingual group whose lexicon was found to be rather balanced in the two languages.

Several other studies have investigated a different set of lexical parameters in RHL spoken in Canada, Sweden, and the United States. Makarova and Terekhova (2020) analyzed narrative samples of 29 Russian-speaking bilinguals (age 5–6) from Canada and 13 monolinguals from Russia. In addition to the traditional measures of vocabulary development that are also central in the present study (TNW and NDW), the authors provide a qualitative analysis of the bilinguals’ vocabulary and their non-canonical lexical forms. The bilingual-monolingual comparison in this study revealed no differences in narrative length in words, different lexemes, words per utterance or speech rate (in number of words per minute). However, RHL speaking children produced significantly more non-canonical lexical forms (e.g., *dyrka* “hole” instead of *nora* “burrow”) as compared to their monolingual peers. Qualitatively, the vocabulary of RHL speaking children and Russian monolinguals had some similar features, such as occasionalisms (i.e., the use of words and word forms invented by children), substitutions of more specific words for more generic ones, and the use of colloquial/vernacular forms. At the same time, some specific features associated with the development of heritage language in immigrant minority settings were also identified, such as the use of dialectal sound constituents of words and code-switches to English. Numerous lexical errors were also observed in Swedish-Russian (*n* = 20, age 6–8) (Ringblom and Dobrova, 2019) and English-Russian (*n* = 15, 4;0–10;11, mean age = 8;3) bilinguals (Bar-Shalom and Zaretsky, 2008). A qualitative analysis of the errors attested in Ringblom and Dobrova (2019) showed that they were largely similar to the errors produced by Russian monolinguals, but they persisted at much later ages in bilinguals (age 6 and later vs. age 3 in monolinguals). In the production of the bilinguals, the lexical errors were largely direct translations from English (Bar-Shalom and Zaretsky, 2008). Accompanied by numerous morphosyntactic errors, these non-target-consistent forms were in stark contrast to the low number of aspectual errors, suggesting that grammatical aspect may be spared from restructuring in RHL and that the lexicon is more vulnerable. This is particularly noticeable during the years in which HSs’ input and dominance are undergoing a major shift in favor of the majority language.

The studies reviewed in this section and especially the studies on RHL spoken in Germany reveal some general tendencies of lexical development in child bilingualism, such as age-appropriate vocabulary growth during preschool years followed by a likely stagnation in primary school as well as a possibility of a shift toward the societal majority language around the age of 5. These tendencies are addressed in the present investigation, although a direct comparison with the reviewed studies is not possible due to the differences in the research methodologies.

## 3. The present study

### 3.1. Research questions

To obtain a more detailed and comprehensive picture of HSs’ lexical development, we investigate lexical production patterns in a large dataset from pre- and primary-school children acquiring RHL in three national contexts–Germany, Norway, and the UK. The diversity of the socio-cultural contexts and the wide age range of our participants should allow us to capture the effects for lexical development associated with the shift in input and dominance of bilinguals. While the main objective of the present study is to investigate lexical development in the HL, additional insights are obtained from a comparison of bilinguals with age-matched Russian monolinguals as well as from a comparison of lexical production in both of the bilinguals’ languages.

We ask the following research questions:

RQ1: How does lexical development proceed in RHL of pre- and primary-school children?

RQ2: How does the shift in language input and use during school-age affect lexical development in RHL, if at all? More specifically, are there signs of stabilization or stagnation of vocabulary development?

RQ3: Which individual background factors can explain the variance in lexical knowledge in the oral narratives of RHL speaking children?

In our previous studies, we have identified several background factors characteristic of RHL bilingualism in Germany, Norway, and the UK, including the child’s age, age of onset of acquisition of the majority language, family type (Russian only or mixed), presence of an older sibling, age of preschool start, size of the HR community, current exposure to HR instruction, and main language of instruction (Mitrofanova et al., 2018; Rodina et al., 2020). Several of these factors have been found to be significant predictors of the bilinguals’ performance in a series of gender assignment tasks, such as language exposure in the home defined in terms of family type (HR family vs. mixed family), the size of the HR community, and current exposure to HR instruction. Furthermore, we have identified that the probability of developing a reduced gender system was predicted in particular by family type, age of preschool start, and current exposure to HR instruction. Overall, addressing the effects of a wide range of factors in the current study will contribute to creating a more precise profile of RHL within and across different national contexts. More specifically, we hypothesize that several of the individual background factors may predetermine the (time of) the dominance shift. Previous research suggests that the shift in language dominance toward the societal language takes place at around age 5. Furthermore, there may be several shifts taking place at different times/ages in different national contexts. As presented in the next section, for Russian-speaking children in Germany and the UK, the onset of regular exposure to the majority language is considerably later than for Russian-speaking children in Norway: children in Norway typically start preschool already at age one, while in Germany and the UK they normally do not start daycare or preschool until the age of 3. Hence, the length of uninterrupted exposure to Russian is shorter for Russian-speaking children in Norway, who also on average receive fewer hours of Russian instruction. Thus, the patterns of lexical development in RHL in Norway, Germany, and the UK may be different, reflecting the input and language dominance patterns in a specific national context.

### 3.2. Participants

The participants in the present study are 143 typically developing pre- and primary-school-aged children (mean age = 6.5), divided into four groups: English-Russian, Norwegian-Russian, German-Russian bilinguals, acquiring Russian as a HL, and Russian monolinguals. All the children attended public preschools, starting at age 1 in Norway, and ages 3–4 in Germany and England. All the bilinguals were attending heritage language classes, with different number of hours of instruction in Russian (varying between two and eight h per week). An overview of the participant groups is presented in Table 1.

**TABLE 1:** Background information on the participants per group and family type.

Group	Total *N*	Mixed family	Minority family	Preschool	Primary school	Age range (mean)
German-Russian	67	19	48	39	28	3–11 (6.7)
Norwegian-Russian	26	13	13	9	17	4–10 (6.7)
English-Russian	19	10	9	1	18	4–10 (7.2)
Russian monolinguals	31	–	–	16	15	4–10 (6.4)

The Norwegian group in this study consisted of 26 typically developing Norwegian-Russian children aged 4–10 (mean age = 6.7) from Tromsø (*n* = 2), Oslo (*n* = 13) and Asker (*n* = 11). Half of the children (*n* = 13, mean age = 6.3) were from mixed Norwegian-Russian households (i.e., families with one Russian- and one Norwegian-speaking parent), the other half from Russian-speaking families (*n* = 13, mean age = 7.1). Nine children attended preschool, and 17 went to public schools with instruction in Norwegian. All the children produced narratives in both Russian and Norwegian.

Sixty-seven German-Russian bilingual children (mean age = 6.7) were recruited in Berlin (*n* = 17), Singen (*n* = 39), and Stuttgart (*n* = 7). Of these, 19 children were from families with one Russian- and one German-speaking parent (mean age = 6.5), while 47 children (mean age = 6.1) were from families with two Russian-speaking parents. Thirty-nine children attended preschool, and 28 went to German primary schools. From these children, we elicited sixty-seven narratives in the HL and fifty-three in the societal language.

In England, 19 English-Russian bilinguals (mean age = 7.2) participated in the study. The narratives were collected in London (*n* = 10) and Reading (*n* = 9). Ten of the children (mean age = 7.2) were raised in families with one Russian- and one English-speaking parent, and nine children (mean age = 7.2) were from families with two Russian-speaking parents. Eighteen out of 19 participants were primary school children in the UK (note that children typically start school after their fourth birthday). The children produced 19 narratives in the HL and 12 narratives in the societal language.

In addition, the narratives of the Russian-speaking monolinguals (*n* = 31, mean age = 6.4) were collected in Ivanovo, Central Russia: 16 children went to preschool and 15 attended primary school.

### 3.3. Methodology

The languages samples were obtained using the Multilingual Assessment Instrument for Narratives (MAIN, Gagarina et al., 2012). MAIN was designed to assess narrative skills (comprehension and production) in multilingual preschool and school-aged children up to the age of ten. The task contains four stories, each with a six-picture sequence: “Dog,” “Cat,” “Baby Goats,” and “Baby Birds.” The stories have parallel plots (in terms of characters, objects, events, foreground and background information) and are controlled for cognitive and linguistic complexity as well as cultural appropriateness.

Two MAIN stories were used to elicit oral narratives – “Baby Goats” and “Baby Birds.” The bilingual participants were divided into two groups, one was presented with “Baby Goats” in Russian and “Baby Birds” in the majority language, while the other did the opposite, “Baby Birds” in Russian and “Baby Goats” in the majority language. Half of the monolingual participants were presented with “Baby Birds,” while the other half were presented with “Baby Goats.” The picture material was printed out and presented according to the MAIN guidelines. The bilingual children were tested on two different days: one session per language with approximately one week in between. Prior to the narrative elicitation, there was a warm-up session when participants listened to a pre-recorded “Dog” or “Cat” story and answered some comprehension questions afterward. This was done in order to create a natural atmosphere and provide an example of storytelling. During the narrative production, the children were asked to choose a story in one of three envelopes and narrate it for the interlocutor without showing the pictures.

The storytelling was recorded and transcribed orthographically, and the transcripts were checked by two experienced researchers. Non-words, mazes, hesitations, repetitions, irrelevant comments, and codeswitching were excluded from the analysis. To investigate lexical production, we used two measures: total number of words (TNW), as a measure of narrative length, and number of different words (NDW), as a measure of lexical diversity. These measures have been used in many studies investigating lexical knowledge in mono- and bilingual children acquiring different languages (e.g., Uccelli and Páez, 2007; Simon-Cereijido and Gutiérrez-Clellen, 2009; Bedore et al., 2010) as well as studies focusing on typical vs. impaired language development (e.g., Watkins et al., 1995; Klee et al., 2004; Hewitt et al., 2005). For typically developing bi- and monolingual children, TNW and NDW are shown to systematically increase with age from preschool to primary school. NDW also tends to be a more sensitive measure than TNW and a better indicator of language growth, since it reflects diversity of vocabulary. In studies of children with language impairment, NDW is found to be consistently lower than that of typically developing peers (e.g., Watkins et al., 1995). For Spanish-English bilinguals in Uccelli and Páez (2007), TNW failed to capture meaningful developmental changes, while NDW was found to be a sensitive measure, since the bilinguals’ lexical diversity increased significantly by age in one of their languages (English). Similarly, NDW increased by grade and was significantly associated with literacy outcomes of Spanish-English bilinguals in Miller et al. (2006). In our own research, NDW was found to be a better predictor of bilinguals’ sensitivity to grammatical gender cues in RHL than TNW (Mitrofanova et al., 2018; Rodina et al., 2020).

A different line of research has been concerned with the reliability of the two lexical productivity measures for comparing mono- and bilingual vocabulary knowledge in storytelling across typologically distant languages: Based on the MAIN narratives of Croatian-Italian bilinguals (*n* = 30, age range 5–7), Hržica and Roch (2021) compare and validate the ability of TNW and NDW as well as the so-called model-based measures to adequately reflect bi- and monolingual children’s lexical abilities.^2^ It is shown that TNW and NDW are able to detect similarities and differences in bi- and monolingual performance as well as performance between languages in bilingual speakers, despite the fact that they are highly sensitive to variability in sample size and language-specific features (morphological richness, diversity of functional words and word segmentation principles). TNW and NDW are also shown to effectively predict bilinguals’ receptive vocabulary scores for each language measured by PPVT.

The present study further contributes to the validation of the TNW and NDW measures in assessing bilingual children’s lexical development.

## 4. Results

The means, standard deviations, and ranges for the two measures for all participant groups are presented in Table 2. In Russian, all bilingual groups show lower TNW and NDW than their monolingual peers. The German-Russian group scores the highest among the bilinguals in both pre- and primary-school subgroups. For all participant groups, the means for TNW and NDW improve with age, but the increase is considerably smaller for the Norwegian-Russian group, which is particularly clear in the primary school subgroup, where they score much lower on both measures. Note, that there was only one child of preschool age in the English-Russian group, which makes these results difficult to compare to the rest of the sample. The means for TNW and NDW are higher in the societal than in the heritage language for all bilingual groups. The German-Russian and Norwegian-Russian groups perform similarly on both measures and the English-Russian school-aged children produce the highest scores.

**TABLE 2:** Means, standard deviations, and ranges for the total number of words (TNW) and number of different words (NDW) for German-Russian, Norwegian-Russian, English-Russian, and Russian monolingual children.

Measures	German-Russian mean (SD), range		Norwegian-Russian mean (SD), range		English-Russian mean (SD), range		Russian monolinguals mean (SD), range	
	Preschool	School	Preschool	School	Preschool	School	Preschool	School
TNW Russian	47.97 (20.27), 14–107	69.21 (30.48), 6–135	36.1 (23.66), 5–88	45.75 (15.03), 25–73	27 (NA), 27–27	68.94 (24.7), 30–120	60.5 (18.42), 24–101	74 (21.87), 52–142
NDW Russian	28.08, (9.44), 12–55	35.79 (12.52), 5–58	22.9 (14.36), 4–53	28 (8.49), 17–44	20 (NA), 20–20	35 (12.01), 13–64	31.62 (6.11), 20–44	40.93 (9.41), 30–71
TNW societal	65.27 (27.39), 12–127	102.56 (33.11), 46−201	66.62 (22.01), 31–99	102.27 (29.13), 61–180	–	142.45 (74.98), 30–296	–	–
NDW societal	30.58 (10.57), 10–53	44.74 (9.94), 27–70	32.88 (6.96), 20–41	45.4 (10.38), 33–74	–	50.18 (16.41), 18–73	–	–

**FIGURE 1:**
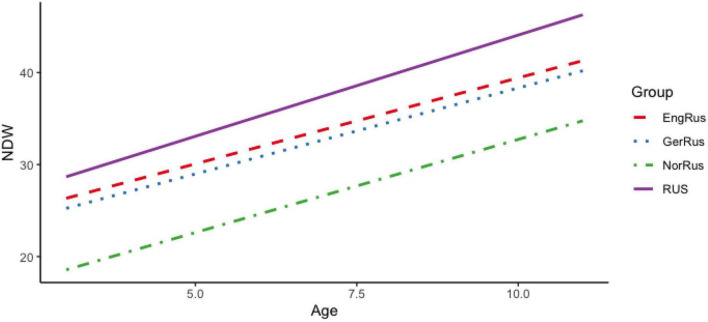
Number of different words (NDW) per narrative in Russian heritage language (RHL) as a function of age and country.

In what follows, we focus on the analysis of lexical development patterns in RHL and the factors that may explain them. The statistical analysis of lexical development in RHL revealed that age and preschool start both had significant effects on the development of the NDW (*p* = 0.002 and *p* = 0.01, respectively) and the TNW (*p* = 0.009 and *p* = 0.001, respectively).^3^ Figure 1 represents the change in the NDW by country with age as a continuous variable. As evident from the figure, NDW increases with age in all groups. At the same time, we can also see that Russian monolinguals score the highest, followed by participants from Germany and the UK, who performed similarly to each other. Participants from Norway produced the lowest NDWs as a group. Recall that the preschool start varies per country, with children in Norway starting preschool at age 1, while children in Germany and the UK typically start at age 3–4.^4^

**FIGURE 2:**
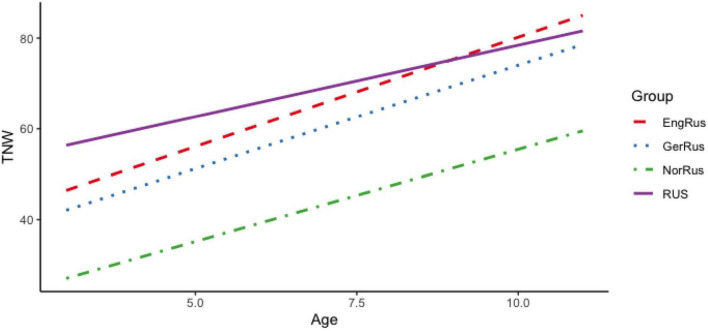
Total number of words (TNW) per narrative in Russian heritage language (RHL) as a function of age and country.

Turning now to our measure of narrative length, the TNW, Figure 2 illustrates changes in this measure with age by country. As evident from the figure, the TNW also increases with age for all participant groups. Interestingly, the participants from Germany and the UK seem to catch up with the monolingual Russian-speaking children in narrative length, while the bilingual children from Norway consistently produce shorter narratives.

**FIGURE 3:**
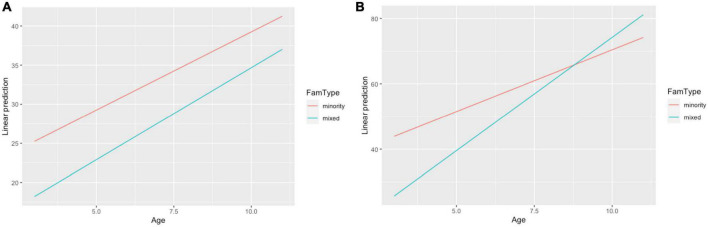
Linear trends for the development of number of different words (NDW) **(A)** and total number of words (TNW) **(B)** as a function of age and family type in Russian heritage language (RHL).

Another variable that has been shown to significantly predict bilinguals’ language development in the HL is family type, i.e., whether both or only one of the parents uses the HL when speaking with the child (Unsworth, 2013; Rodina and Westergaard, 2017; Mitrofanova et al., 2018). To analyze the results statistically, we ran a linear regression analysis where two lexical variables (NDW and TNW) were predicted as an interaction of family type (mixed vs. minority) and age, with preschool start as an independent predictor. *Post hoc* comparisons of estimated marginal trends confirmed a significant effect of age for children from mixed as well as minority language families, for both the NDW (*p* = 0.002 for both family types) and the TNW (*p* = 0.0001 for mixed and *p* = 0.01 for minority language families). Figures 3A, B illustrate these linear trends for the NDW and the TNW, respectively as predicted by the models. The statistical analysis and the figures demonstrate that children from mixed families exhibit a steeper developmental change in the overall narrative length (TNW) and eventually catch up with the children from minority language families (3b). At the same time, the development of the NDW measure proceeds in parallel for children from mixed and minority language families (3a).

**FIGURE 4:**
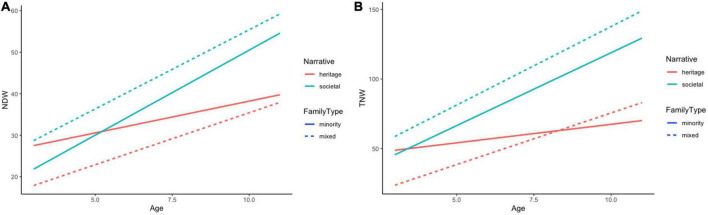
Development of number of different words (NDW) **(A)** and total number of words (TNW) **(B)** in the societal and heritage language as a function of age and family type.

Finally, we also compared the dynamics of narrative development in both the heritage and the societal language of the bilinguals. Figures 4A, B summarize the effects of age on the two narrative indices in the two languages by family type (mixed vs. minority families). To compare the dynamics statistically, we ran a linear regression analysis where the two narrative indices were predicted as a three-way interaction of family type (mixed vs. minority), language (heritage vs. societal), and age. The analysis revealed a significant interaction of age and language for both indices, the NDW (*p* = 0.015) and the TNW (*p* = 0.006), suggesting that the difference between the narrative skills in the two languages becomes significantly larger with age (indicating steeper development in the societal as compared to the HL).

## 5. Discussion

In the (Section “3.1. Research questions”) we asked the following research questions, which we now discuss in turn:

RQ1: How does lexical development proceed in RHL of pre- and primary-school children?

RQ2: How does the shift in language input and use during school-age affect lexical development in RHL, if at all? More specifically, are there signs of stabilization or stagnation of vocabulary development?

RQ3: Which individual background factors can explain the variance in lexical knowledge in the oral narratives of RHL speaking children?

The results displayed in Table 2 and Figures 1, 2 may be used to answer RQ1. Lexical development in RHL is characterized by a clear and steady increase with age for both of our main lexical productivity measures, TNW and NDW. That is, the children go through a gradual and even development with respect to the TNW and NDW used in their narratives. In the preschool years for both measures, the differences between the monolinguals and the three groups of RHL speakers are relatively constant throughout development, with the Russian monolinguals having the highest rate of lexical diversity, the Norwegian-Russian group the lowest, and the RHL speakers in Germany and the UK in the middle. During primary school, all bilingual groups show age sensitive development for both measures, but while the RHL children in Germany and the UK catch up with the monolinguals, the children from Norway perform significantly lower. The higher lexical productivity of the German-Russian and English-Russian children may be attributed to their later start of the preschool (at around the age of 3) and hence a later onset of regular exposure to the societal language, as compared with the Norwegian-Russian bilinguals. We return to this issue in connection to RQ3. Not surprisingly, the comparison of lexical development in RHL and in the societal language in Table 1 and Figure 4 reveals an advantage of the latter, but the differences are not dramatic, and importantly, there is parallel development from pre- to primary-school in both languages. Furthermore, it is clear that there is considerable individual variation not only in RHL, but also in the societal language in all participant groups. Overall, the growth of expressive vocabulary that we observe in the preschool years is similar to the one reported in Czapka et al. (2021). However, in contrast to Montanari et al.’s (2020) results, there is no stagnation in the vocabulary growth in RHL of our participants from Germany or other countries. With the differences in the methodologies in mind (picture-naming in Montanari et al. (2020) vs. oral narratives in the current study), a possible explanation could be that, at the onset of primary school, the Russian-speaking 6-year-olds in Montanari et al. (2020) had a larger vocabulary than the same-age bilinguals in the present study. As a result, the vocabulary growth may appear more pronounced in the current data sample.

The observed developmental trajectory is yet unexpected, since in connection with RQ2 we predicted to see a certain reduction or stagnation in the lexical development of the RHL speakers at the onset of primary school. While this is of course a very positive state of affairs for the RHL children involved, it is somewhat surprising, considering the emphasis that is put on the change in language dominance in HL research. In fact, this dominance shift is often part of the definition of what a HL speaker is (e.g., Montrul, 2016). So why do we not see a stagnation in our HL data? One speculation is that our findings could be due to the fact that this is a cross-sectional study, not a longitudinal one. Thus, there may be some self-selection in the type of speakers who have participated in our study. That is, the oldest children that we have recruited are HL children who have continued learning Russian (both at home and in HL instruction), and who have therefore felt confident about participating in our study. There is of course a possibility that there are many other RHL children at this age who have not reached this level of Russian. But even so, our findings suggest that a drop in lexical diversity in the HL is not a necessary consequence of a dominance shift resulting from starting school and thus an increase in exposure to the majority language. We return to this issue below when we discuss the effects of family type on lexical productivity.

Our data show that the individual variation among our RHL children is substantial (Table 2). Our RQ3 asks what factors may account for this variation in lexical diversity among the RHL children across different countries. In addition to age (which is clearly a significant factor, the children are gradually increasing their lexical diversity with time), the timing of start in preschool is an important factor, as also found in our previous work (Mitrofanova et al., 2018; Rodina et al., 2020) as well as in previous work on RHL in Germany where larger vocabulary size in RHL was found to correlate with later age of onset of German (Czapka et al., 2021). This accounts for the Norwegian-Russian children having a lower lexical diversity in the HL from the very beginning of language acquisition, a situation that persists throughout childhood. As mentioned above, children growing up in Norway generally start preschool already at age 1, which means that they have massive exposure to the majority language even in their pre-linguistic stage. In contrast, Russian HL children in Germany and the UK normally do not start preschool until the age of three, which means that they have ample time to develop the lexical and grammatical skills of the HL before being exposed to large proportions of the majority language.

Another important factor is family type, i.e., whether the children grow up with one or two Russian-speaking parents. Figure 4A shows that children growing up in homes where they get mixed input generally score lower on the NDW measure for the HL than the children who are only exposed to Russian at home, while they score higher on the majority language. Importantly, the children from mixed-input families also score better on the majority language than the HL, and this is a situation that increases over time. In fact, this measure indicates that the dominance shift should only occur in the development of the children with two Russian-speaking parents, since the children who get input from both languages in the home (i.e., a mixed family type) are dominant in the majority language from the very beginning and throughout development. This also means that the stagnation that we expected to see in Figures 1, 2 is somehow concealed by the fact that the data of children from different family types are mixed in those graphs. When the data are separated by family type as in Figure 4A, we see that lexical diversity development in the HL slows down considerably compared to the lexical development of the majority language, but only for the children who grow up with two Russian-speaking parents. That is, the lexical development in the majority language has a much steeper slope than the slope for the HL, which only shows a slight increase over time. The fact that there is positive development in the majority language, especially in children where both parents are speakers of Russian, highlights the importance of HL preservation in the family context which ensures HL maintenance in child bilinguals, and, at the same time, does not hinder development in the societal language. A similar conclusion has been reached in Gagarina et al. (2014) based on the evidence from German-Russian and Hebrew-Russian bilinguals and there are also studies where positive interaction between HL vocabulary skills and L2 vocabulary acquisition has been found (e.g., Grøver et al., 2018).

In line with previous research, including recent studies on vocabulary acquisition in RHL (e.g., Klassert et al., 2014), we also see in Figure 4 that the two lines for HL and majority language development cross around age 5 for the children from families with two Russian-speaking parents, indicating that the dominance shift occurs already at this young age in this group of RHL children. This finding is compatible with the reversed accentedness pattern that we observed in an earlier study with the same group of German-Russian bilinguals, where the incidence of a perceived foreign accent decreased from younger (preschool) to older (primary school) children in German, while it increased for Russian (Kupisch et al., 2021). While no such shifts have been attested in our previous studies investigating the acquisition of grammatical gender in RHL in the same participant groups, it is clear that all three linguistic domains are affected by input factors, with language exposure in the home in terms of family type (HR family vs. mixed family) and age of starting preschool as the major predictors. Overall, the results from lexical, grammatical, and phonological acquisition in pre- and primary-school bilinguals seem to support the view that having longer exclusive or uninterrupted exposure to a HL in early childhood is beneficial for HL development and outcomes (cf., Bar-Shalom and Zaretsky, 2008; Lloyd-Smith et al., 2020). At the same time, it is not straightforward from our dataset whether lexical development is more susceptible to input factors than grammatical development. Our in-depth investigation of gender assignment in RHL in a large data sample of bilinguals from five different national contexts–Germany, Norway, the UK, Latvia, and Israel–showed that the bilingual children were sensitive to morphophonological cues for gender assignment, although they were less target-like than monolinguals (Mitrofanova et al., 2018, 2022; Rodina et al., 2020). Further examination of the bilinguals’ individual profiles showed that while the masculine-feminine-neuter distinction was present in the majority of bilinguals across all countries (174/211, 83%), there was still a certain number of children (37/211, 17%) who had difficulties acquiring neuter or grammatical gender altogether. Taken together, the developmental patterns from lexical and grammatical acquisition in RHL can be used to conclude that variation is an inherent characteristic of the heritage speaker population.

Finally, the results of the present study contribute to an ongoing debate as to what extent lexical productivity measures, such as TNW and NDW, reflect general lexical knowledge of bilinguals. Our results suggest that both of these narrative productivity measures are sensitive indicators of lexical development and are able to detect developmental patterns across typologically different languages of bilingual speakers. Both measures increased significantly by age, but the measure of lexical diversity (NDW) was also able to detect a shift in lexical development in the group of bilinguals from the HL families (Figure 4A). Thus, corresponding to previously made observations, there is a tendency for NDW to be a more sensitive measure than TNW in the current data sample. Overall, we can conclude that the general lexical knowledge of bilinguals can be reliably established based on relatively short and variable samples of spoken narratives, presenting potential for overall bilingual language assessment, especially of languages for which (adequate) assessment materials are unavailable.

The current study has several limitations which are likely to affect our ability to fully and objectively explore lexical development in HSs. Our participants were recruited and tested in different Russian language centers where they received additional HL support. Therefore, despite considerable variation in performance, our sample may be biased toward motivated and proficient HL learners. Ideally, we should have included bilinguals who do not receive additional HL support. This would also provide further insights about the role of HL education or lack of thereof on bilinguals’ language development. Furthermore, the sample sizes of the three participant groups varied greatly and were rather small for the Norwegian-Russian and especially the English-Russian group, where there were also no preschool children. Methodologically, the study could have benefited from including other lexical measures and tasks (e.g., picture-naming) which would have allowed a more direct comparison of lexical development in RHL across studies. Given the diversity of the HL communities within and across different national contexts, future research conducted in new HL communities will likely advance the discussion of the impact of the socio-linguistic environment on HL development and maintenance.

## 6. Conclusion

In this paper, we have analyzed lexical development in three populations of Russian heritage children growing up in Norway, Germany, and the UK, comparing both the heritage and the majority language of the bilingual children. Furthermore, a comparison is made with monolingual children growing up in Russia. Data have been collected using the elicitation material in the Multilingual Assessment Instrument for Narratives (MAIN), from which two lexical measures have been extracted, total number of words (TNW) and number of different words (NDW), measuring narrative length and lexical diversity, respectively. Results show that there is a gradual increase in both measures in both languages of the bilinguals, but that the bilinguals generally score lower than the monolinguals in Russian, and the bilinguals from Norway score considerably lower than the heritage children in Germany and the UK. The latter finding is explained by the early exposure to the majority language in Norway, as most children start daycare at age one, while children in Germany and the UK do not start until age three or later. Indications of stagnation or dominance shift in the heritage language is only visible in the narratives of children with two Russian-speaking parents, as the children from mixed families are dominant in the majority language already from early on. Our results corroborate findings from other studies on heritage language children, showing that speaking a heritage language has no adverse effects on the development of the majority language.

## Data availability statement

The raw data supporting the conclusions of this article will be made available on request to the corresponding author.

## Ethics statement

The studies involving human participants were reviewed and approved by the Norwegian center for research data. Written informed consent to participate in this study was provided by the participants’ legal guardian/next of kin.

## Author contributions

NM, YR, and MW carried out the collection of data with bilingual participants. YR carried out the collection of control data with monolingual Russian children and had the main responsibility for finalizing the draft. NM, AB, and YR are responsible for data transcription and analysis. All authors wrote the manuscript and are responsible for the conception and design of the study.

## Footnotes

1 In Rodina et al. (2020), we also report on data from Hebrew-Russian and Latvian-Russian bilinguals. These data are not included in the present study, since lexical measures were not obtained from those groups.

2 Model-based measures applied in Hržica and Roch (2021) were measure D, moving average type-token ratio, and hypergeometric diversity of D.

3 All analyses were performed using R Statistical Software (v4.0.3; R Core Team, 2021).

4 It is possible to start preschool at age 1 in Germany and the UK; however, none of our participants reported starting preschool earlier than age 3.

## References

[B1] Bar-Shalom E. G. Zaretsky E. (2008). Selective attrition in Russian-English bilingual children: Preservation of grammatical aspect. *Int. J. Biling.* 12 281–302. 10.1177/1367006908098572

[B2] Bedore L. M. Pena E. D. Gillamb R. B. Hoa T. H. (2010). Language sample measures and language ability in Spanish English bilingual kindergarteners. *J. Commun. Disord.* 43 498–510. 10.1016/j.jcomdis.2010.05.002 20955835 PMC2958172

[B3] Bialystok E. Luk G. Peets K. F. Yang S. (2010). Receptive vocabulary differences in monolingual and bilingual children. *Bilingualism* 13 525–531. 10.1017/S1366728909990423 25750580 PMC4349351

[B4] Brehmer B. Kurbangulova T. (2017). “Lost in transmission? Family language input and its role for the development of Russian as a heritage language in Germany,” in *Integration, identity and language maintenance in young immigrants. Russian Germans or German Russians*, eds Isurin L. Riehl C. M. (Amsterdam: John Benjamins), 225–268. 10.1075/impact.44.c8

[B5] Czapka S. Topaj N. Gagarina N. (2021). A four-year longitudinal comparative study on the lexicon development of Russian and Turkish heritage speakers in Germany. *Languages* 6:27. 10.3390/languages6010027

[B6] Dieser E. (2009). *Genuserwerb im Russischen und Deutschen: Korpusgestützte Studie zu ein- und Zweisprachigen Kindern und Erwachsenen. [The Acquisition of Gender in German and Russian: A Corpus-Based Study with Mono- and Bilingual Children and Adults.].* München: Sagner. 10.3726/b12030

[B7] Dunn L. M. Dunn D. M. (2007). *Peabody picture vocabulary test (PPVT-4)*, 4th Edn. Minneapolis, MN: Pearson. 10.1037/t15144-000

[B8] Gagarina N. Armon-Lotem S. Altman C. Burstein-Feldman Z. Klassert A. Topaj N. (2014). “Age, input quantity and their effect on linguistic performance in the home and societal language among Russian-German and Russian-Hebrew preschool children,” in *The challenges of diaspora migration interdisciplinary perspectives on Israel and Germany*, eds Silbereisen R. K. Titzmann P. F. Shavit Y. (Farnham: Ashgate Publishing), 63–82.

[B9] Gagarina N. Fichman S. Galkina E. Protassova E. Ringblom N. Rodina Y. (2021). “How oral texts are organized in monolingual and heritage Russian. Evidence from six countries,” in *Language Impairment in Multilingual Settings. LITMUS in action across Europe [Trends in language acquisition research, 29]*, eds Armon-Lotem S. Grohmann K. K. (Amsterdam: John Benjamins), 47–76. 10.1075/tilar.29.02gag

[B10] Gagarina N. Klop D. Kunnari S. Tantele K. Välimaa T. Balciuniene I. (2012). Multilingual assessment instrument for narratives (MAIN). *ZAS Pap. Linguist.* 56 1–140.

[B11] Gathercole V. C. M. Thomas E. M. (2009). Bilingual first-language development: Dominant language takeover, threatened minority language take-up. *Bilingualism* 12 213–237. 10.1017/S1366728909004015

[B12] Grøver V. Lawrence J. Rydland V. (2018). Bilingual preschool children’s second-language vocabulary development: The role of first-language vocabulary skills and second-language talk input. *Int. J. Biling.* 22 234–250. 10.1177/1367006916666389

[B13] Hewitt L. E. Hammer C. S. Yont K. M. Tomblin J. B. (2005). Language sampling for kindergarten children with and without SLI: Mean length of utterance, IPSYN, and NDW. *J. Commun. Disord.* 38 197–213. 10.1016/j.jcomdis.2004.10.002 15748724

[B14] Hržica G. Roch M. (2021). “Lexical diversity in bilingual speakers of Croatian and Italian,” in *Language impairment in multilingual settings: LITMUS in action across Europe*, eds Armon-Lotem S. Grohmann K. (Amsterdam: John Benjamins), 100–129. 10.1075/tilar.29.04hrz

[B15] Jia R. Paradis J. (2015). The use of referring expressions in narratives by Mandarin heritage language children and the role of language environment factors in predicting individual differences. *Bilingualism* 18 737–752. 10.1017/S1366728914000728

[B16] Klassert A. Gagarina N. Kauschke C. (2014). Object and action naming in Russian- and German-speaking monolingual and bilingual children. *Bilingualism* 17 73–88.

[B17] Klee T. Stokes S. F. Wong A. Fletcher P. Gavin W. J. (2004). Utterance length and linguistic diversity in Cantonese-speaking children with and without language impairment. *J. Speech Lang. Hear. Res.* 47 1396–1410. 15842018 10.1044/1092-4388(2004/104)

[B18] Krüger I. (2021). *Gender agreement patterns in heritage Russian. Ph.D. dissertation.* Humboldt University of Berlin.

[B19] Kupisch T. Kolb N. Rodina Y. Urek O. (2021). Foreign accent in pre-and primary school heritage Bilinguals. *Languages* 6:96.

[B20] Laleko O. (2010). *The syntax-pragmatics interface in language loss: Covert restructuring of aspect in heritage Russian. Doctoral dissertation.* Minneapolis, MN: University of Minnesota.

[B21] Laleko O. (2022). Word order and information structure in heritage and L2 Russian: Focus and unaccusativity effects in subject inversion. *Int. J. Biling.* 26 749–766.

[B22] Lloyd-Smith A. Bayram F. Iverson M. (2020). “The effects of heritage language experience on lexical and morphosyntactic outcomes,” in *Studies in Turkish as a heritage Languages*, ed. Bayram F. (Amsterdam: John Benjamins), 63–86.

[B23] Makarova V. Terekhova N. (2020). Russian-as-a-heritage-language vocabulary acquisition by bi-/multilingual children in Canada. *Russ. Lang. Stud.* 18 409–421. 10.22363/2618-8163-2020-18-4-409-421

[B24] Meir N. Janssen B. (2021). Child heritage language development: An interplay between cross-linguistic influence and language-external factors. *Front. Psychol.* 12:651730. 10.3389/fpsyg.2021.65173 34867570 PMC8632657

[B25] Miller J. Heilmann J. Nockerts A. Iglesias A. Fabiano L. Francis D. J. (2006). Oral language and reading in bilingual children. *Learn. Disabil. Res. Pract.* 21 30–43.

[B26] Mitrofanova N. Rodina Y. Urek O. Westergaard M. (2018). Bilinguals’ sensitivity to grammatical gender cues in Russian: The role of cumulative input, proficiency, and dominance. *Front. Psychol.* 9:1894. 10.3389/fpsyg.2018.0189 30364150 PMC6193086

[B27] Mitrofanova N. Urek O. Rodina Y. Westergaard M. (2022). Sensitivity to microvariation in bilingual acquisition: Morphophonological gender cues in Russian heritage language. *Appl. Psychol.* 43 41–79.

[B28] Montanari E. Abel R. Tschudinovski L. Graßer B. (2020). “Vocabulary development in the heritage languages Russian and Turkish between ages 6 and 10,” in *Lost in Transmission: The role of attrition and input in heritage language development*, eds Brehmer B. Treffers-Daller J. (Amsterdam: Benjamins), 151–170.

[B29] Montrul S. (2016). *The acquisition of heritage languages.* Cambridge: CUP.

[B30] Otwinowska A. Meir N. Ringblom N. Karpava S. La Morgia F. (2021). Language and literacy transmission in heritage language: Evidence from Russian-speaking families in Cyprus, Ireland, Israel and Sweden. *J. Multiling. Multicult. Dev.* 42 357–382. 10.1080/01434632.2019.1695807

[B31] Pearson B. Z. Ferñandez S. C. Oller D. K. (1993). Lexical development in bilingual infants and toddlers: Comparison to monolingual norms. *Lang. Learn.* 43 93–120. 10.1111/j.1467-1770.1993.tb00174.x

[B32] Pearson B. Z. Ferñandez S. C. Lewedeg V. Oller D. K. (1997). The relation of input factors to lexical learning by bilingual infants. *Appl. Psychol.* 18 41–58. 10.1017/S0142716400009863

[B33] Polinsky Maria. (2008). Gender under incomplete acquisition: heritage speakers’ knowledge of noun categorization. *Heritage Lang. J.* 6 40–71. 10.46538/hlj.6.1.3

[B34] R Core Team (2021). *R: A language and environment for statistical computing.* Vienna: R Foundation for Statistical Computing.

[B35] Ringblom N. Dobrova G. (2019). Holistic constructions in heritage Russian and Russian as a second language: Divergence or delay? *Scando Slav.* 65 94–106. 10.1080/00806765.2019.1586577

[B36] Rodina Y. (2017). Narrative abilities of preschool bilingual Norwegian-Russian children. *Int. J. Biling.* 21 617–635. 10.1177/1367006916643528

[B37] Rodina Y. Westergaard M. (2017). Grammatical gender in bilingual Norwegian-Russian acquisition: The role of input and transparency. *Bilingualism* 20 197–214.

[B38] Rodina Y. Kupisch T. Meir N. Mitrofanova N. Urek O. Westergaard M. (2020). Internal and external factors in heritage language acquisition: Evidence from heritage Russian in Israel, Germany, Norway, Latvia and the UK. *Front. Educ.* 5:20. 10.3389/feduc.2020.00020

[B39] Schwartz M. Minkov M. Dieser E. Protassova E. Moin V. Polinsky M. (2015). Acquisition of Russian gender agreement by monolingual and bilingual children. *Int. J. Biling.* 19 726–752. 10.3389/fpsyg.2018.01894 30364150 PMC6193086

[B40] Scontras G. Fuchs S. Polinsky M. (2015). Heritage language and linguistic theory. *Front. Psychol.* 6:1545. 10.3389/fpsyg.2015.01545 26500595 PMC4598584

[B41] Sheng L. Lu Y. Kan P. F. (2011). Lexical development in Mandarin-English bilingual children. *Bilingualism* 14 579–587.

[B42] Silvén M. Voeten M. Kouvo A. Lundén M. (2014). Speech perception and vocabulary growth: A longitudinal study of Finnish-Russian bilinguals and Finnish monolinguals from infancy to three years. *Int. J. Behav. Dev.* 38 323–332. 10.1177/0165025414533748

[B43] Simon-Cereijido G. Gutiérrez-Clellen F. V. (2009). A cross-linguistic and bilingual evaluation of the interdependence between lexical and grammatical domains. *Appl. Psychol.* 30 315–337. 10.1017/S0142716409090134 19444336 PMC2681320

[B44] Uccelli P. Páez M. M. (2007). Narrative and vocabulary development of bilingual children from kindergarten to first grade: Developmental changes and associations among English and Spanish skills. *Lang. Speech Hear. Serv. Sch.* 38 225–236. 10.1044/0161-1461(2007/024) 17625049 PMC2881826

[B45] Unsworth S. (2013). Current issues in multilingual first language acquisition. *Annu. Rev. Appl. Linguist.* 33 21–50.

[B46] Watkins R. V. Kelly D. J. Harbers H. M. Hollis W. (1995). Measuring children’s lexical diversity: Differentiating typical and atypical language learners. *J. Speech Hear. Res.* 34 1134–1141. 10.1044/jshr.3806.1349 8747826

[B47] Yan S. Nicoladis E. (2009). Finding le mot juste: Differences between bilingual and monolingual children’s lexical access in comprehension and production. *Bilingualism* 12 323–335.

